# circRNA disease: a manually curated database of experimentally supported circRNA-disease associations

**DOI:** 10.1038/s41419-018-0503-3

**Published:** 2018-04-27

**Authors:** Zheng Zhao, Kuanyu Wang, Fan Wu, Wen Wang, Kenan Zhang, Huimin Hu, Yanwei Liu, Tao Jiang

**Affiliations:** 1 Beijing Neurosurgical Institute, Beijing, 100050 China; 2 Department of Neurosurgery, Beijing Tiantan Hospital, Capital Medical University, Beijing, 100050 China; 3 Department of Neurosurgery, The Second Affiliated Hospital of Soochow University, Suzhou, 215123 China; 4 Centre of Brain Tumor, Beijing Institute for Brain Disorders, Beijing, 100069 China; 5 China National Clinical Research Centre for Neurological Diseases, Beijing, 100050 China

Dear Editor(s),

Circular RNAs (termed as circRNAs), as a large family of noncoding RNA molecules, have been reported to be transcribed in eukaryotes^1,2^. With advances of high-throughput sequencing technologies and bioinformatics methods, a number of circRNAs are identified and related data is accumulating rapidly, including annotation, expression profiles, and biological functions. More recently, lots of studies have revealed that circRNA dysfunctions are associated with a broad range of diseases, including cancers^3,4^, neurodegeneration and cerebrovascular diseases^5^. Therefore, circRNAs might be a novel type of potential biomarkers or treatment targets for disease prognosis and therapy. However, a public resource of high-quality curated disease-associated circRNAs remains unavailable. Here, we have developed circRNA disease that provides a user-friendly interface for searching disease-associated circRNAs. The database is freely available at http://cgga.org.cn:9091/circRNADisease/.

To obtain the high confident experimentally supported circRNA-disease associations, all circRNA-disease entries were manually curated from PubMed database using the keywords “circRNA disease”, “circular RNA disease”, “circRNA cancer” and “circular RNA cancer” that had been recorded before November 2017 from the National Center for Biotechnology Information (Supplementary Information). Different researchers were assigned to double-check all circRNA-disease pairs. In total, circRNA disease documents 354 curated relationships between 330 circRNAs and 48 diseases. Each entry in the circRNA disease includes detailed information on a circRNA-disease association, including circRNA ID or name, disease name, the circRNA expression pattern, experimental detection techniques, circRNA-associated partners, a brief description of circRNA biological function, literature references, other annotation information, etc.

The circRNA disease database provides a user friendly, open access web interface that allows users to browse, search, and upload the circRNA-disease association in the database (Fig. 1). In the “Browse” page, users can browse circRNA-disease associations by circRNA ID, circRNA name, or disease name of interest. For each of entering circRNA or disease, circRNA disease will show a list of matched entries. As an essential component, circRNA disease provides the “Search” allowing users to quickly retrieve detailed information on each circRNA-disease association. Here, user can query the database through circRNA ID/name, circRNA host gene, and disease name. The “Search” page provides also a fuzzy search function, facilitating smart assistant by listing the closest entries to that expectation. In addition to exist circRNA-disease associations, we also encourage users to submit novel experimentally supported relationship through the “Submit” page. Once approved by the submission review committee, we will update database. Moreover, all the datasets in the circRNA disease can be downloaded freely for this community and researchers. Finally, a detailed tutorial for the usage of this database is provided in the “Help” page.

**Fig. 1:**
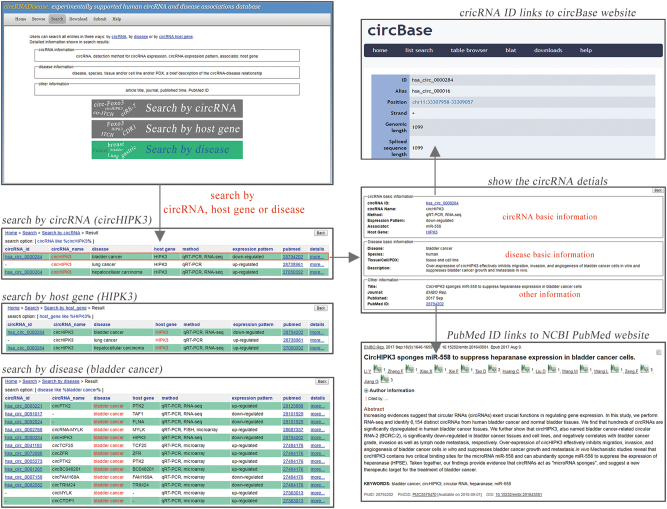
A schematic workflow of circRNADisease

In conclusion, circRNA disease may serve as an immeasurable resource for understanding the roles of circRNAs in diseases. The features of circRNA disease contain: (i) quickly browse circRNA-disease associations with literature evidence; (ii) systematically search circRNA-disease relationships by circRNA, circRNA host gene, disease; and (iii) all the circRNA-disease pairs can be downloaded freely. The circRNA disease will continue to update the experimentally supported circRNA-disease association data per 3 months. Meanwhile, novel bioinformatic tools will be developed for further analyzing circRNA-disease associations.

## Electronic supplementary material

Supplementary information
