# Supplementary material for: circRNA disease: a manually curated database of experimentally supported circRNA-disease associations
Source: Cell Death Dis. 2018 Apr 27;9(5):475. doi: 10.1038/s41419-018-0503-3 (PMC5919922; doi:10.1038/s41419-018-0503-3)

**Supplementary information**

**circRNADisease: a manually curated database of experimentally supported circRNA-disease associations**

Zheng Zhao^1, 6^, Kuanyu Wang^1, 6^, Fan Wu^1, 6^, Wen Wang^2, 3^, Kenan Zhang^1^, Huimin Hu^1^, Yanwei Liu^2^, Tao Jiang^1, 2, 4, 5, *^

^1^Beijing Neurosurgical Institute, Beijing 10050, China

^2^Department of Neurosurgery, Beijing Tiantan Hospital, Capital Medical University, Beijing 10050, China.

^3^Department of Neurosurgery, The Second Affiliated Hospital of Soochow University, Suzhou 215123, China

^4^Centre of Brain Tumor, Beijing Institute for Brain Disorders, Beijing 100069, China.

^5^China National Clinical Research Centre for Neurological Diseases, Beijing 100050, China.

^6^Equal contribution

*Corresponding to [taojiang1964@163.com](mailto:taojiang1964@163.com)

**Section I: Database Content**

In recent years, more and more papers focused on circRNA research have been published. Since 2016, researchers have attempted to investigate the circRNA dysfunction in diseases (Supplementary Figure 1a). In this article, more than 800 published literature were systematically reviewed, and manually collected 354 associations between 330 circRNAs and 48 diseases, of which cancer (54%, 26/48) and cardiovascular and cerebrovascular diseases (27%, 13/48) (Supplementary Figure 1b). The circRNADisease database provides a user-friendly, open access interface for an easy query of each entry by circRNA ID or name, disease name. Moreover, we provided useful links to other databases, including circBase and GeneCards. We also provided the hyper link to NCBI PubMed database together with an official PubMed ID as well as complete citation.

Finally, all data in circRNADisease had been organized using MySQL 14.14, a professor database management system. The Web site was developed based on JSP using a Servlet framework, and is deployed on a Tomcat 6.0.44 web server, and runs under a CentOS 5.5 system. The database is available at http://cgga.org.cn:9091/circRNADisease. The circRNADisease was fully tested in Google Chrome (version 62 and later).

**Section II: Database Update Plan**

Recently, the emergence of high-throughput technologies, such as RNA-seq, microarray, single cell sequencing, have provided an unprecedented opportunity to capture the circRNA expression in disease. The circRNAs are emerging as novel potential molecules for diseases diagnosis, treatment and prognosis. At present, only a small set of circRNAs were being reported in published literature, however, they may act pioneers to greatly promote the understanding of circRNAs in diseases. Increasing evidence has shown that dysfunctions of circRNAs are associated with a wide range of diseases. These advances in this field will greatly promote to further extend circRNADisease. The circRNADisease will continue to update the experimentally supported circRNA-disease association data per three months. Meanwhile, novel bioinformatic tools will be developed for further analyzing circRNA-disease associations. We hope that circRNADisease may serves as an immeasurable resource for understanding the roles of circRNAs in diseases.

**Supplementary Figure 1**

**Statistics of published circRNA-disease paper. a) Number of** **published circRNA-disease paper per quarter.** Number of circRNA-disease paper published in each quarter from 2015 to 2017. The ‘other’ in some year means that the month of publication is unknown. **b) Disease statistics in published circRNA-disease paper.** The distribution of diseases from published circRNA-disease association.


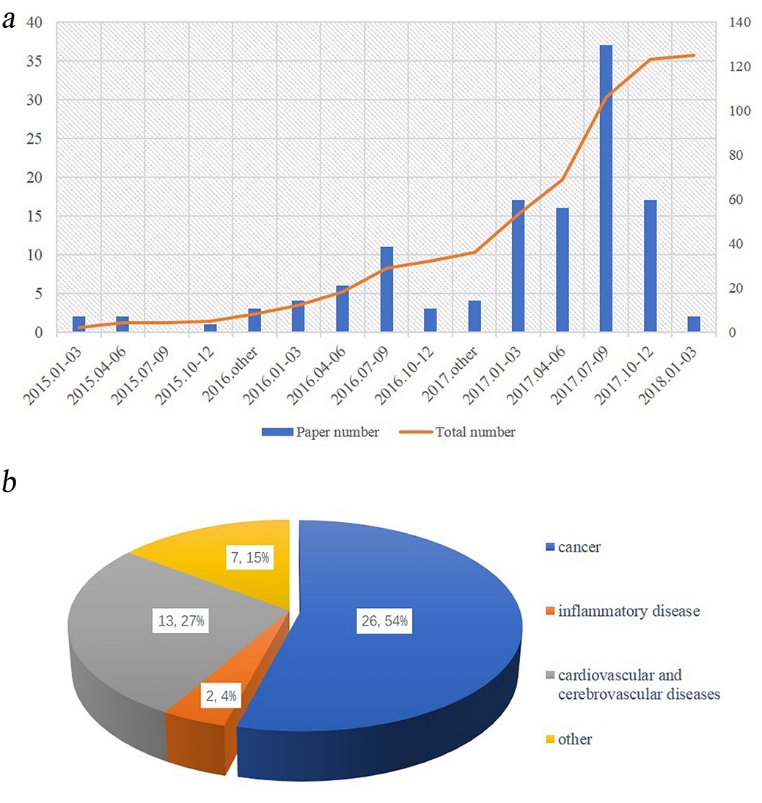

Supplement: Supplementary file 1 — Supplementary information [file 41419_2018_503_MOESM1_ESM.docx]
